# The first RT-qPCR confirmed case of tick-borne encephalitis in a dog in Scandinavia

**DOI:** 10.1186/s13028-020-00550-2

**Published:** 2020-09-10

**Authors:** Elina Andersson, Anna Kendall, Angelika Url, Angelika Auer, Michael Leschnik

**Affiliations:** 1 Section of Pathology, Department of Biomedical Sciences and Veterinary Public Health, Faculty of Veterinary Medicine and Animal Science, Swedish University of Agricultural Sciences (SLU), Uppsala, Sweden; 2 Institute of Pathology, Department of Pathobiology, University of Veterinary Medicine, Vienna, Austria; 3 Institute of Virology, Department of Pathobiology, University of Veterinary Medicine, Vienna, Austria; 4 Small Animal Clinic, Department for Companion Animals, University of Veterinary Medicine, Vienna, Austria

**Keywords:** Canine, Histopathology, Immunohistochemistry, Meningoencephalomyelitis, Necropsy, Pathology, Serology

## Abstract

**Background:**

Tick-borne encephalitis (TBE) is a zoonotic neurological disease caused by tick-borne encephalitis virus (TBEV), a flavivirus endemic in parts of Europe and Asia. Seroconversion without signs of clinical disease is common in dogs and most of the cases previously described have been tentatively diagnosed by combining neurologic signs with serum antibody titres. Here, the first Scandinavian RT-qPCR-confirmed clinical case of TBE in a dog is reported.

**Case presentation:**

A 4-year old castrated male Pointer Labrador cross was presented with acute-onset ataxia. During hospitalisation, the dog developed seizures. Despite aggressive treatment with steroids, antimicrobials and sedation/anaesthesia, there was continued deterioration during the following 24 h after admission and the dog was euthanised and submitted for necropsy. Histopathological changes in the brain were consistent with lymphoplasmacytic and histiocytic meningoencephalomyelitis. RT-qPCR examination of the brain was positive for TBEV, confirming infection.

**Conclusions:**

Meningoencephalomyelitis caused by TBEV should be a diagnostic consideration in dogs presenting with clinical signs of central nervous system disease such as acute-onset ataxia and seizures in areas where TBEV-positive ticks are endemic. Clinical TBE may be underdiagnosed in dogs due to lack of specific testing.

## Background

Tick-borne encephalitis (TBE) is a zoonotic neurological disease, caused by tick-borne encephalitis virus (TBEV) that belongs to the genus flavivirus and family *Flaviviridae* [1]. The virus is endemic in parts of Asia and Europe. In Scandinavia, TBEV is endemic in Sweden, Finland and parts of Denmark and Norway [1, 2] and is of major health concern in humans. In Europe, approximately 3000 human cases were reported annually between 2000 and 2010 [2]. In Sweden, TBE case numbers in humans have increased during the last decade [3], from 174 cases in 2010 to a total of 358 cases in 2019, with a corresponding incidence of 3.46 per 100,000 inhabitants [3]. The highest incidence is around the Mälaren Valley in the region of Uppsala, reaching 9.12 per 100,000 inhabitants in 2019 [3].

The European subtype of TBEV is transmitted mainly by the tick vector *Ixodes ricinus,* which is active between spring and autumn [1, 4–6]. This is the main tick vector responsible for disease transmission in Sweden [1, 7]. *Ixodes persulcatus*, the tick vector for the Far Eastern- and Siberian subtype of TBEV [1], was discovered for the first time in Sweden in 2015 [8]. Out of 276 *Ixodes persulcatus* ticks collected in 2015–2016, all were negative for TBEV [9]. The TBEV prevalence in the Swedish *Ixodes ricinus* tick population varies depending on study, tick stage and geographical location, ranging from 0.10 to 4.48% [10].

In dogs, acute manifestation of TBE takes a monophasic course with an estimated incubation period of 5–9 days [11, 12]. Clinical signs include fever and neurological abnormalities such as altered behaviour and consciousness, neck pain, hyperaesthesia, proprioceptive dysfunction, paresis, ataxia and seizures and well as cranial- and spinal nerve deficits [11–13]. Peracute and acute cases lead to death within a week [11]. Fatal outcome is reported to occur in 16–50% of clinical cases of TBE in dogs [12, 14, 15]. The main pathological finding comprises lymphoplasmacytic and histiocytic inflammation in the cerebrum, meninges, brain stem and spinal cord [11, 13, 15–17]. In humans, the clinical symptoms and pathologic findings are similar to those in dogs, except for a biphasic course of disease [1, 18–21].

Only a few clinical cases of TBEV infection in dogs have been previously reported, occurring in Switzerland, Austria, Germany, Italy and Sweden [13, 22]. The first reported suspected Swedish canine TBE case was in 1960; a 5-year-old female Irish setter which developed neurological signs after exposure to ticks in the Stockholm archipelago [23]. Two more cases were described in 2001 and 2007 respectively [24, 25]. All of the previous Swedish cases have only been tentatively diagnosed by measuring serum antibody titres [23–25]. As fever and neurological signs occur early in the disease course in dogs, antibody response to TBEV infection may not be detectable in peracute and acute cases, where death may occur within a week [11, 12]. In addition, seroconversion without clinical signs is common in dogs [11, 13, 15, 26]. A rising antibody titre, although indicative of TBE in association with neurological signs, therefore does not provide a definitive diagnosis.

More reliable in vivo diagnostic methods for TBE include polymerase chain reaction (PCR) examination of blood or cerebrospinal fluid (CSF) during the febrile, viraemic phase [11, 27, 28]. Post-mortem, PCR may be conducted on brain tissue and viral antigen may be detectable by immunohistochemical examination [11, 13, 16]. The following report describes the first Scandinavian real-time polymerase chain reaction (RT-qPCR) confirmed case of clinical disease of TBE in a dog.

## Case presentation

A 4-year old, 25 kg castrated male Pointer Labrador cross was admitted to the University Companion Animal Hospital at the Swedish University of Agricultural Sciences in Uppsala in late August 2019 due to acute-onset ataxia. 24 h prior to admission the dog had been mildly obtunded and had shown mild discomfort on palpation of the thoracic spine. A positive response was seen after one dose of firocoxib (4.5 mg/kg *per os*) and the following morning the dog was bright and alert but showed a single episode of mild proprioceptive deficits of the right hindlimb. There was deterioration during the day, and, in the afternoon, ataxia of all four limbs but mentation and appetite remained normal. The dog was from a rural area in the Mälaren Valley with access to forest walks and occasional attached ticks had been noted and removed throughout the summer. The dog had been treated with a single dose of fluralaner spot on (Bravecto^®^, Intervet, Sweden) approximately 3 months prior to presentation.

On presentation the patient was very stressed and showed obvious ataxia with mild bilateral forelimb hypermetria. Cranial nerve functions were within normal limits. Palpation of the thoracic spine elicited a mild pain response, but there was no obvious pain on manipulation and palpation of the cervical spine. Rectal temperature was 39.1 °C. Cardiac auscultation could not be adequately performed due to heavy panting but mucous membranes were pink and moist. Abdominal palpation was normal. Serum biochemistry showed normal levels of liver enzyme activities and creatinine. A total blood count (CBC) was within normal limits but C-reactive protein (CRP) was mildly elevated at 14.3 mg/L (0–10 mg/L).

The dog was hospitalised and remained stable over night with good appetite and normal urination. The following morning during routine blood sampling (approximately 15 h after admission), the patient rapidly deteriorated. The ataxia worsened to the point where the dog could no longer remain ambulatory. The breathing turned into heavy panting and there was facial grimacing with tense, retracted lips indicating involvement of the facial nerve and brain stem. Opisthotonus was noted but normal palpebral reflexes and pupillary light reflexes were still present bilaterally. Pain response and withdrawal reflex was present in all four limbs.

Sedation was achieved with medetomidine (4 µg/kg), and subsequent medetomidine constant rate infusion (CRI) at 1 µg/kg/h. Due to the case being admitted during a weekend with limited access to laboratory and other diagnostic modalities, no additional examinations were performed. As there was a clinical suspicion of meningoencephalitis, intravenous application of prednisolone acetate (2 mg/kg) and doctacillin (21 mg/kg) was initiated. Despite an initial response to sedation, approximately 2 h after initiation of the medetomidine CRI, seizure-like activity with mild paddling of the forelimbs was noted. Midazolam was added to the CRI protocol at 0.17 mg/kg/h and the medetomidine CRI increased to 2 µg/kg/h. Despite this, seizures could not be controlled and the dog was therefore switched to a propofol CRI at 6 mg/kg/h. The dose had to be gradually increased during the following hours. Despite addition of midazolam CRI and increasing propofol CRI up to 30 mg/kg/h the seizures could not be controlled. Overnight, the dog deteriorated with poor oxygenation and occasional episodes of apnea. When attempts were made to decrease the propofol CRI, the seizures recurred. Due to continued deterioration the dog was euthanised (approximately 30 h after admission) and submitted for necropsy.

Gross findings included two bilateral asymmetrical areas of haemorrhage in the superficial cortex of the frontal lobe of the brain, one in each hemisphere, measuring 0.5 cm in diameter. The brain was diffusely congested and slightly oedematous. No other gross findings were observed. The brain and spinal cord were fixed in 10% neutral buffered formalin, routinely processed and stained with haematoxylin–eosin (HE). Sections of frontal, parietal and temporal telencephalon, thalamus, hippocampus, basal nuclei, mesencephalon, cerebellum and brain stem as well as the cervical, thoracal and lumbal portion of the spinal cord were histologically evaluated.

**Fig. 1:**
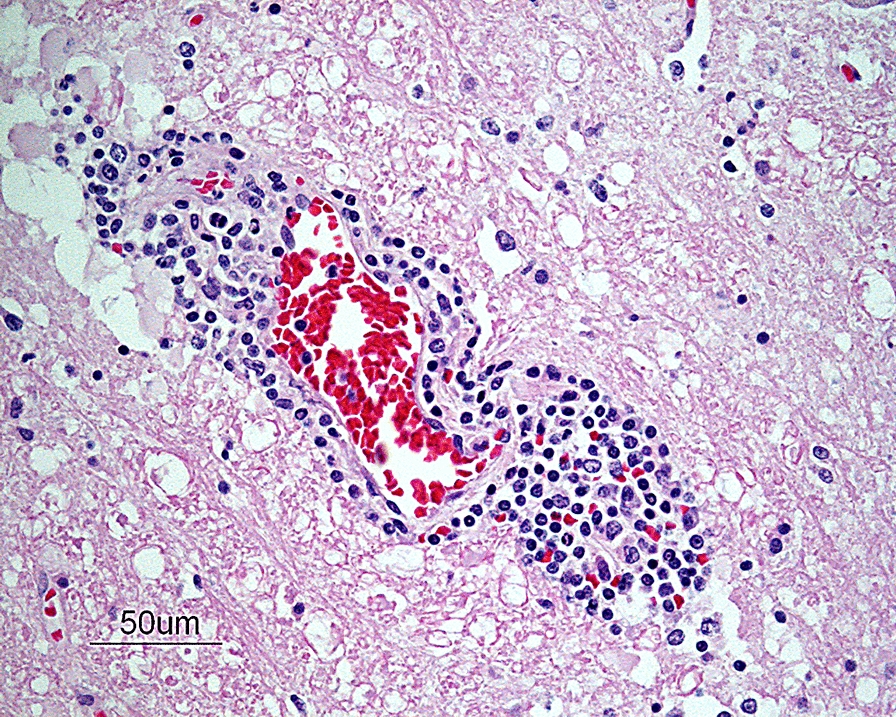
Photomicrograph showing a perivascular cuff consisting of lymphocytes, plasma cells and histiocytes in the brain stem. HE-stain. Bar: 50 µm

**Fig. 2:**
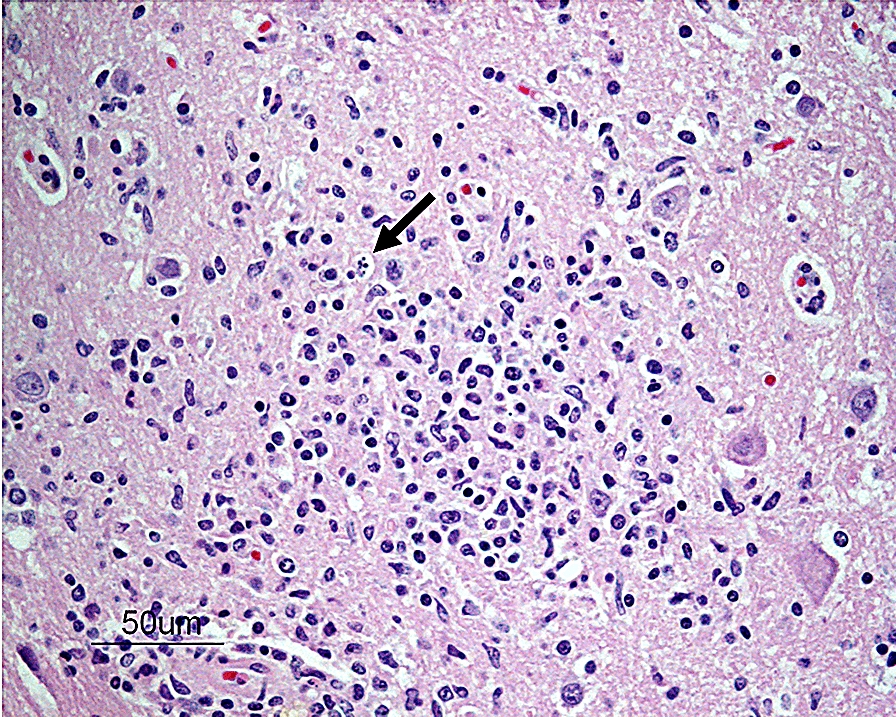
Photomicrograph showing an area of diffuse gliosis with single cell necrosis (arrow) in the neuroparenchyma at the level of the mesencephalon. HE-stain. Bar: 50 µm

**Fig. 3:**
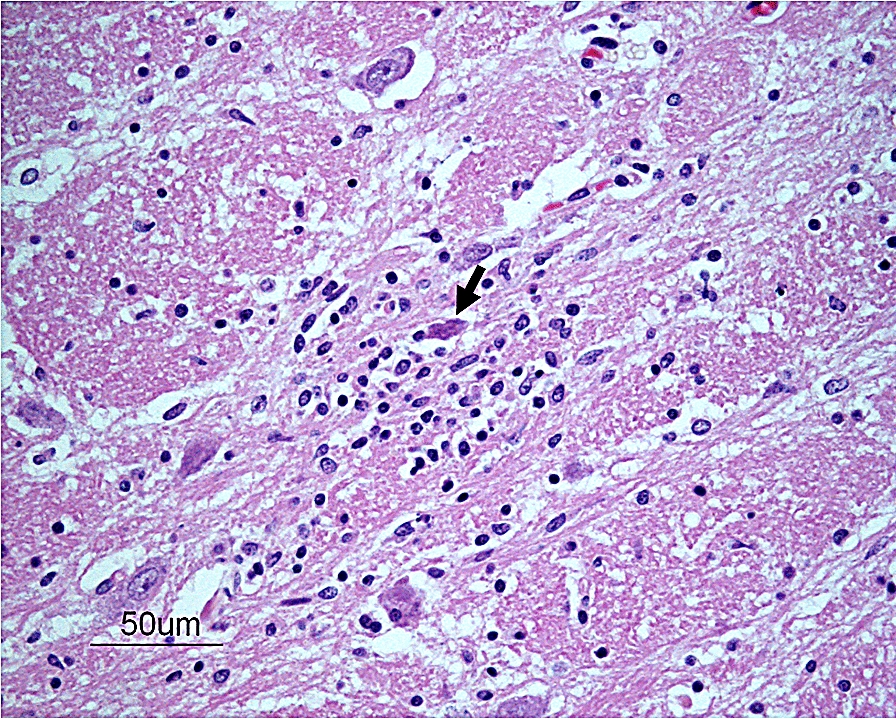
Photomicrograph showing a necrotic neuron (arrow) surrounded by glial cells (interpreted as neuronophagia) in the thalamus. HE-stain. Bar: 50 µm

On microscopic examination, all sections of the brain and spinal cord showed multifocal to coalescing moderate to severe inflammatory infiltrates composed of lymphocytes, plasma cells and histiocytes, located within the neuroparenchyma and surrounding vessels as up to 10-layer thick perivascular cuffs (Fig. 1). In the brain, inflammation was seen in both white and grey matter, meanwhile in the spinal cord, inflammation was more pronounced in the grey matter of both dorsal and ventral horns. Most severe inflammatory changes were seen in the areas of thalamus, mesencephalon, brain stem and all segments of spinal cord. Multifocally, neuroparenchyma of both brain and spinal cord showed prominent nodular to diffuse gliosis with single cell necrosis (Fig. 2) as well as neuronal degeneration and microglia surrounding neuronal debris (interpreted as neuronophagia) (Fig. 3). The neuroparenchyma displayed mild to moderate rarefaction. Within the leptomeningeal subarachnoid space and surrounding leptomeningeal vessels in both brain and spinal cord were multifocal to coalescing infiltrates of mild to moderate numbers of lymphocytes, plasma cells and occasional histiocytes. No inclusion bodies were found. Three brain sections representing mesencephalon, cerebellum and brain stem were stained with Giemsa, with no additional findings. The histopathological changes were consistent with a lymphoplasmacytic and histiocytic meningoencephalomyelitis.

Immunohistochemical investigation of brain tissue using a polyclonal rabbit antibody to TBEV (strain “Hochosterwitz, kindly provided by Prof. K. Stiasny, Dept. Virology, MedUni Vienna, Austria; dilution 1:500), was performed automatically on an autostainer (Lab Vision AS 360, Thermo Fisher Scientific, Waltham, MA, USA). Antigen retrieval was performed on deparaffinized and rehydrated sections by pronase digestion. Endogenous peroxidase activity was blocked by incubation in H_2_O_2_, while background reduction was achieved by application of UV Protein Block (Thermo Scientific, Freemont, CA, USA). For detection of the primary antibody a secondary poly-HRP-goat anti-rabbit-IgG (BrightVision Poly-HRP-Anti Rb, Immunologic, VB Duiven, NL) was used followed by visualisation with DAB (DAB Quanto Substrate System, Thermo Scientific, Freemont, CA, USA). Subsequently, all sections were counterstained with haematoxylin, dehydrated and mounted. No specific staining for TBE could be noted in the sections. Brain sections from a mouse with experimental TBE served as positive control. For negative control, the primary antibody was omitted.

Paraffin-embedded brain tissue was submitted for RT-qPCR examination. The tissue was deparaffinised using xylene and ethanol washes, thereafter tissue was digested by using tissue lysis buffer and Proteinase K (Qiagen, Hilden, Germany). For nucleic acid extraction, 140 µL of tissue lysate was used employing QIAamp Viral RNA Mini QIAcube Kit using QIAcube (Qiagen) according to the manufacturer’s instructions. TBEV-specific sequences were detected by an RT-qPCR developed by Schwaiger et Cassinotti [27], with primers F-TBE 1: 5′-GGG CGG TTC TTG TTC TCC-3′, R-TBE 1: 5′-ACA CAT CAC CTC CTT GTC AGA CT-3′ and TBE-specific probe TBE-Probe-WT: FAM-5′-TGA GCC ACC ATC ACC CAG ACA CA-3′-BHQ1 [27]. RT-qPCR was carried out in a reaction volume of 20 µL using the qScript One-Step RT-qPCR Kit (Quantabio, Beverly, MA, USA) following the manufacturer’s guidelines. To ensure validity of the assays, positive and negative controls were included. The PCR was positive at a cycle threshold (CT) value of 30, confirming TBEV infection.

## Discussion and conclusions

The examination of the brain and spinal cord in this case revealed a lymphoplasmacytic and histiocytic meningoencephalomyelitis caused by infection with TBEV, as confirmed by RT-qPCR. An alternative method for confirmation of clinical TBEV infection in vivo in dogs includes PCR examination of serum or CSF [11, 28]. However, TBE viral nucleic acid is only detectable in serum during the initial viraemic phase, which may correspond to a period when clinical signs are not yet fully exhibited [11, 28]. In addition, viraemia does not necessarily lead to clinical disease and altogether this may make in vivo diagnosis by PCR from blood samples difficult in dogs.

In this case, diagnostic testing of brain tissue was performed post mortem and the positive RT-qPCR result confirms TBEV infection. The immunohistochemical examination was negative for TBEV which is probably due to several factors. The method detects antigen without multiplication of the target, and is therefore less sensitive than PCR that targets DNA or RNA with subsequent multifold amplification of the nucleic acid. In the light of a PCR CT value of 30 the amount of viral antigen may also have been too low for detection by immunohistochemistry. Furthermore, rapid immunological viral clearance in the brain and CSF may have caused a false negative immunohistochemical result [16, 29].

In this case, no in vivo testing for TBEV was attempted due to lack of awareness of TBE as a potential differential diagnosis. In the light of the increased TBEV infection incidence in humans and the expansion of endemic areas during the last decades [2, 3], TBE should be considered as a possible differential diagnosis in dogs exhibiting neurological signs.

The main pathological finding in this case, comprising lymphoplasmacytic and histiocytic inflammation within the brain and spinal cord, reflects the clinical signs of meningoencephalomyelitis. The histopathological pattern of neural non-purulent inflammation is in itself unspecific but suggestive of viral infection [17]. Many of the possible histopathological differential diagnoses either comprise viral encephalitidies that do not currently occur in Sweden, such as West Nile virus infection and rabies, or have attributes that do not fit in with the clinical presentation of this case. In addition to this, the histopathological findings were consistent with the changes seen in a retrospective post-mortem examination of brain tissue from eight Austrian dogs with clinical TBE confirmed by immunohistochemistry, except for the absence of glial shrubbery in the cerebellum [16]. In both the Austrian study and this study, changes included lymphohistiocytic meningitis, neuronal necrosis and neuronophagia, gliosis and non-purulent perivascular cuffing in the neuroparenchyma. In this case, the most severe changes were seen in the thalamus, mesencephalon, brainstem and spinal cord, which is in high accordance with the Austrian results.

In conclusion, this case report shows that meningoencephalomyelitis caused by TBEV should be a diagnostic consideration in dogs presenting with clinical signs of neurological disease such as acute-onset ataxia and seizures in areas where TBEV-positive ticks are endemic [2, 3]. In vivo PCR testing of blood or, preferably, CSF should be included as part of the work up of suspected cases. Furthermore, awareness should be raised that in times of climate change and warmer weather, ticks may be active for longer periods of the year [30–32] and clinical cases of TBE in dogs may be encountered during a larger part of the year in endemic areas in the future.

## Data Availability

The datasets used and/or analysed during the current study are available from the corresponding author on reasonable request.

## Abbreviations

CBC: Complete blood count
CRI: Constant rate infusion
CRP: C-reactive protein
CSF: Cerebrospinal fluid
CT: Cycle threshold
DAB: 3′3-Diaminobenzidine
HE: Haematoxylin and eosin
HRP: Horseradish peroxidase
IHC: Immunohistochemistry
PCR: Polymerase chain reaction
RT-qPCR: Real-time polymerase chain reaction
TBE: Tick-borne encephalitis
TBEV: Tick-borne encephalitis virus
